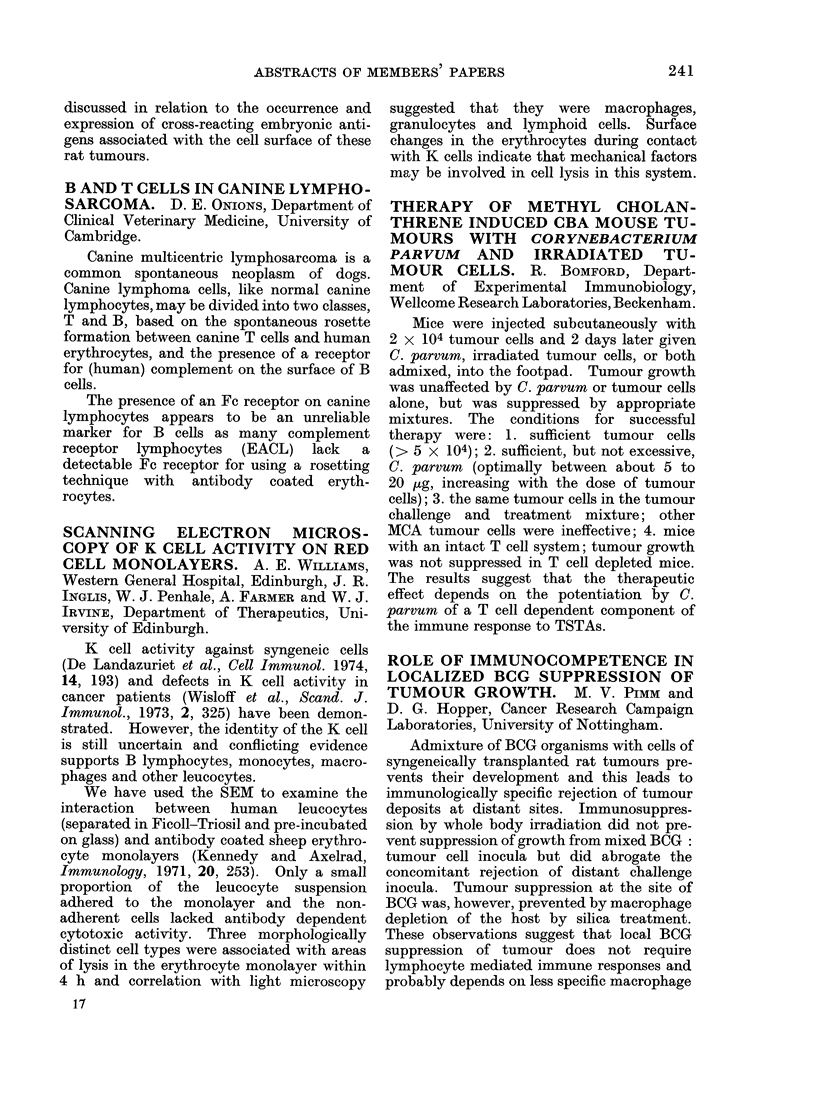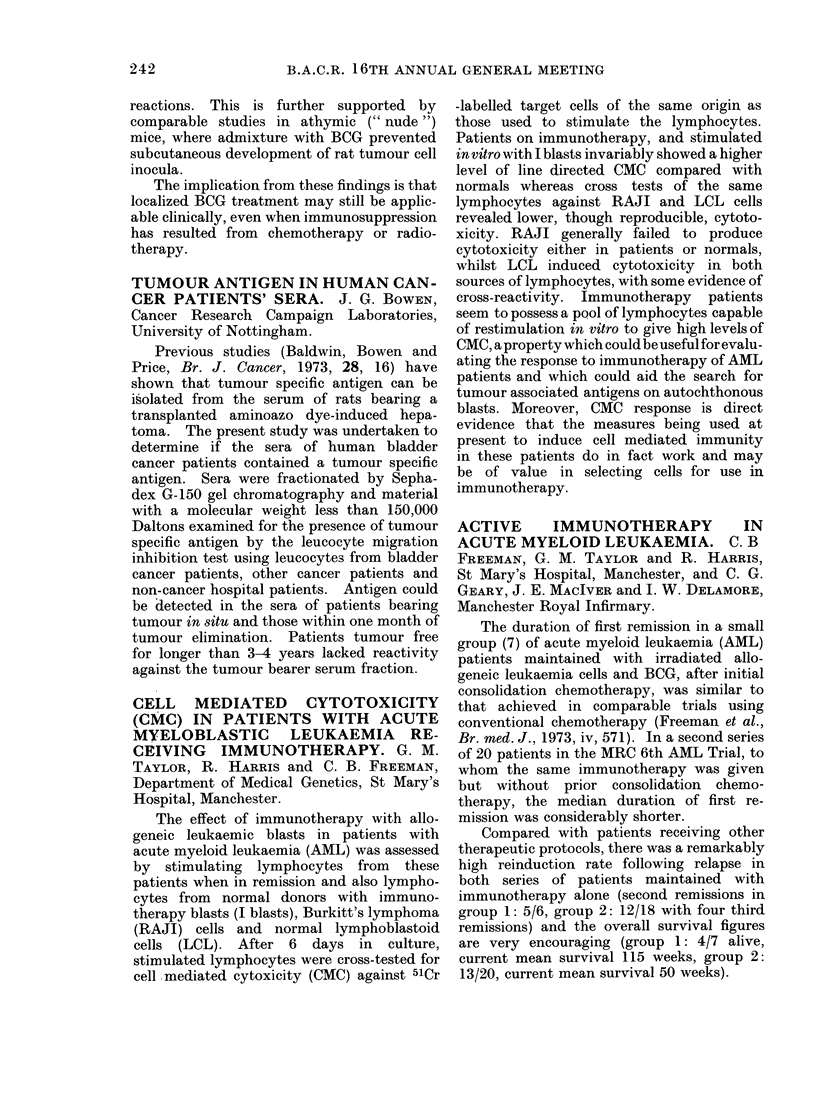# Proceedings: Role of immunocompetence in localized BCG suppression of tumour growth.

**DOI:** 10.1038/bjc.1975.160

**Published:** 1975-08

**Authors:** M. V. Pimm, D. G. Hopper


**ROLE OF IMMUNOCOMPETENCE IN LOCALIZED BCG SUPPRESSION OF TUMOUR GROWTH.** M. V. Pimm and D. G. Hopper, Cancer Research Campaign Laboratories, University of Nottingham.

Admixture of BCG organisms with cells of syngeneically transplanted rat tumours prevents their development and this leads to immunologically specific rejection of tumour deposits at distant sites. Immunosuppression by whole body irradiation did not prevent suppression of growth from mixed BCG : tumour cell inocula but did abrogate the concomitant rejection of distant challenge inocula. Tumour suppression at the site of BCG was, however, prevented by macrophage depletion of the host by silica treatment. These observations suggest that local BCG suppression of tumour does not require lymphocyte mediated immune responses and probably depends on less specific macrophage reactions. This is further supported by comparable studies in athymic ("nude") mice, where admixture with BCG prevented subcutaneous development of rat tumour cell inocula.

The implication from these findings is that localized BCG treatment may still be applicable clinically, even when immunosuppression has resulted from chemotherapy or radiotherapy.